# Genetic variants in *TRPM7* associated with unexplained stillbirth modify ion channel function

**DOI:** 10.1093/hmg/ddz198

**Published:** 2019-08-19

**Authors:** James H Cartwright, Qadeer Aziz, Stephen C Harmer, Sudhin Thayyil, Andrew Tinker, Patricia B Munroe

**Affiliations:** 1 Clinical Pharmacology, William Harvey Research Institute, Barts and the London School of Medicine and Dentistry, Queen Mary University of London, Charterhouse Square, London EC1M 6BQ, UK; 2 School of Physiology, Pharmacology and Neuroscience, Faculty of Life Sciences, The University of Bristol, Biomedical Sciences Building, University Walk, Bristol BS8 1TD, UK; 3 Centre for Perinatal Neuroscience, Imperial College London, London W12OHS, UK

## Abstract

Stillbirth is the loss of a fetus after 22 weeks of gestation, of which almost half go completely unexplained despite post-mortem. We recently sequenced 35 arrhythmia-associated genes from 70 unexplained stillbirth cases. Our hypothesis was that deleterious mutations in channelopathy genes may have a functional effect *in utero* that may be pro-arrhythmic in the developing fetus. We observed four heterozygous, nonsynonymous variants in transient receptor potential melastatin 7 (*TRPM7*), a ubiquitously expressed ion channel known to regulate cardiac development and repolarization in mice.

We used site-directed mutagenesis and single-cell patch-clamp to analyze the functional effect of the four stillbirth mutants on *TRPM7* ion channel function in heterologous cells. We also used cardiomyocytes derived from human pluripotent stem cells to model the contribution of *TRPM7* to action potential morphology.

Our results show that two *TRPM7* variants, p.G179V and p.T860M, lead to a marked reduction in ion channel conductance. This observation was underpinned by a lack of measurable *TRPM7* protein expression, which in the case of p.T860M was due to rapid proteasomal degradation. We also report that human hiPSC-derived cardiomyocytes possess measurable *TRPM7* currents; however, siRNA knockdown did not directly affect action potential morphology.

*TRPM7* variants found in the unexplained stillbirth population adversely affect ion channel function and this may precipitate fatal arrhythmia *in utero*.

## Introduction

Stillbirth is the devastating loss of a fetus after 22 weeks of gestation during pregnancy. In the UK, there were 4.64 stillbirths per 1000 total births in 2013 (1). The majority of these are caused by obstetric complications, placental insufficiency, genetic disorders, infection and umbilical cord anomalies. However, despite extensive post-mortem investigation, 15–45% of stillbirths go completely unexplained (1–3).

Deleterious mutations within ion channel genes can cause severe arrhythmias and are known causes of sudden death in young adults (4). The cause of death is usually found after a negative post-mortem. We recently sequenced a custom panel of 35 arrhythmia-associated genes in 70 unexplained stillbirth cases from the Cardiac Ion Channelopathies in Stillbirth study (5). We found four cases to have putative pathogenic variants in four long QT syndrome genes, *KCNE1, KCNE2, SCN5A* and *KCNJ2*. We subsequently characterized the functional effect of the heterozygous variant (p.R40Q) found in the *KCNJ2* gene. This gene encodes the inwardly rectifying potassium channel Kir2.1, which controls the resting membrane potential and contributes to cardiac repolarization (Phase 3) (6). The p.R40Q mutation led to significantly reduced inward currents compared to wild-type (WT) channels, which would be predicted to have a deleterious effect on the cardiomyocytes ability to regulate its’ membrane voltage. Alongside these data, we observed predicted damaging variants in several other genes, including transient receptor potential melastatin 7 (*TRPM7*)*,* a gene implicated from genome-wide association studies of QT interval (7). Four heterozygous nonsynonymous variants were observed in *TRPM7* in four individual cases.

*TRPM7* is a non-selective cation channel with a serine/threonine kinase domain in the C-terminus and is ubiquitously expressed in mammalian cells (8). Current opinion regards *TRPM7* as a key transporter of divalent cations (Zn^2+^, Mg^2+^ and Ca^2+^) across the cell membrane (9,10), while the α-kinase is capable of phosphorylating downstream effector kinases and modifying chromatin (11,12). *TRPM7* plays a key role in dictating cell proliferation, survival, apoptosis alongside organogenesis and embryonic survival (13–16). A previously reported genetic variant, p.T1482I, has been shown to increase the sensitivity of *TRPM7* to inhibition by Mg^2+^ (17). Temporal control of *TRPM7* expression in the mouse embryo is required for correct cardiac development (15).

The four nonsynonymous variants in *TRPM7* lie within conserved amino acid positions and were predicted to functionally affect the protein by at least one of three predicted software tools (PolyPhen, SIFT or Mutation Taster (18,19)). The first two, p.G179V and p.R494Q, lie within the N-terminal domain of *TRPM7*. The third, p.T860M, is within the second transmembrane domain, whilst the final variant, p.E1205G, resides in the C-terminal domain, upstream of the alpha kinase (Table 1).

**Table 1:** Summary of *TRPM7* variants found in stillbirth cases. We sequenced four predicted damaging variants represented as both cDNA (c.) and protein (p.) changes. Listed are their relative locations within known domains of the *TRPM7* protein. GERP++ score estimates the level of functional constraint that DNA position is under, within coding regions the average conservation score is 2 (28). PolyPhen and SIFT scores rate amino acid substitutions on their probability of impacting protein structure and function by providing values between 0 and 1 (18,19). Variants rated above 0.85 by PolyPhen are confidently predicted to be damaging, while those rated below 0.05 by SIFT are considered likely to be deleterious. Mutation taster analyses substitutions based upon base conservation, specific amino acid substitutions, functional domain location and other possible splicing effects. All *TRPM7* variants were rated as 1 and annotated as disease causing. gnomAD frequency represents the variant’s abundance in the online genome aggregation database project, containing 125 748 exome sequences.

Mutation	Domain	GERP++	PolyPhen	Mutation taster	SIFT	gnomAD frequency
c.G536T p.G179V	N-terminal	4.65	1	1	0	7.48e-5
c.G1481A p.R494Q	N-terminal	4.11	1	1	0.21	1.71e-5
c.C2579T p.T860M	Transmembrane	5.59	1	1	0	7.44e-6
c.A3614G p.E1205G	C-terminal	5.3	0.561	1	0	1.90e-5

In this study, we transfect a *TRPM7* expressing plasmid into HEK293 and CHO-K1 cells and record the resulting current using whole-cell patch-clamp electrophysiology. We demonstrate that three *TRPM7* variants (p.G179V, p.R494Q and p.T860M) significantly alter current densities in comparison to the WT ion channel. This effect was not due to an altered response to magnesium inhibition nor was *TRPM7* transcription perturbed. Instead, we found a lack of protein expression in p.G179V and p.T860M-transfected cells, alongside an increase in p.R494Q protein. Interestingly, inhibiting the 26S proteasome with MG132 treatment increased p.R494Q expression and inhibited p.T860M expression. To further characterize the importance of *TRPM7* in developing cardiac myocytes, we measured *TRPM7* current in hiPSC-derived cardiomyocytes. While we found *TRPM7* expression to increase in the early stages of differentiation, and there is a large outward *TRPM7*-like current, knockdown with *TRPM7* siRNA had no effect on cardiomyocyte action potential morphology.

## Results

### Expressing *TRPM7* in HEK293 and CHO-K1 cells

**Figure 1:**
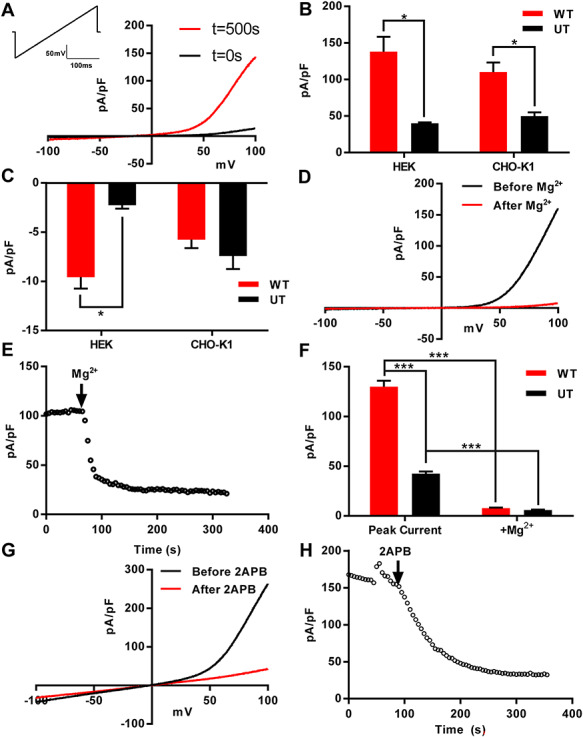
*TRPM7* current recorded from transfected CHO-K1 and HEK293 cells**.** (**A**) Representative *TRPM7* current trace from CHO-K1 cell transfected with *TRPM7* at initial break-in (*t* = 0 s) and after current run-up (*t* = 500 s). (**B**) Current density at +80 mV from both CHO-K1 and HEK293 cells transiently transfected with *TRPM7* (red) compared with untransfected cells (black). (**C**)Matching inward current density from cells in (A), analyzed at −80 mV. (**D**) Whole-cell current trace before (black) and after (red) addition of 10 mm magnesium to extracellular solution during whole-cell recording of a WT *TRPM7*-transfected CHO-K1 cell. (**E**) Time course of CHO-K1 whole-cell current showing immediate inhibition following 10 mm magnesium treatment. (**F**) Summary of whole-cell peak current data and following 10 mm magnesium treatment in both WT *TRPM7*-transfected and UT CHO-K1 cells at +80 mV. (**G**) Whole-cell current trace before (black) and after (red) addition of 50 μM 2-APB to extracellular solution during whole-cell recording of a WT *TRPM7*-transfected CHO-K1 cell. (**H**) Time course of current at +80 mV from cell shown in (G), showing current reduction over 300 s following 2-APB addition. (H) WT, UT = untransfected. ^*^—*P* < 0.05, ^*^^*^^*^—*P* < 0.001.

To ascertain the effect the nonsynonymous mutations may have on *TRPM7* function, we transfected both CHO-K1 and HEK293 cells with a WT *TRPM7* expression plasmid. After 5–10 min of dialysis, large outward currents were detected, with an I–V relationship typical of *TRPM7* (Fig. 1A). We analyzed outward and inward current at +80 and −80 mV, respectively, in both CHO-K1 and HEK293 cells (Fig. 1B and C). Using CHO-K1 cells, we demonstrated that these currents were susceptible to inhibition to 10 mm magnesium (Fig. 1D–F). Current density was also susceptible to inhibition by treatment with 50 μM 2-APB (Fig. 1G and H). HEK293 cell *TRPM7* current was also inhibited by treatment with extracellular magnesium (data not shown). These data confirmed that we had an efficient expression system to test the effect of our stillbirth sequenced variants on *TRPM7* function.

### Variants alter *TRPM7* current density in CHO-K1 and HEK293 cells

**Figure 2:**
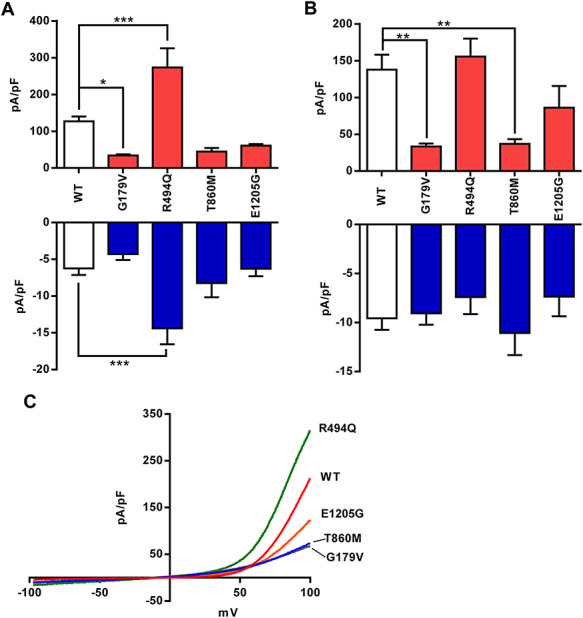
Stillbirth *TRPM7* variants alter current density in a cell-specific manner. (**A**) Summary data of average *TRPM7* current density at +80 mV (red) and −80 mV (blue) from CHO-K1 cells (WT *n* = 28, variant *n* = 13–8). (**B**) Summary data of average *TRPM7* current density at +80 mV (red) and −80 mV (blue) from CHO-K1 cells (WT *n* = 24, variant *n* = 12–7). (**C**) Whole-cell current trace recordings from CHO-K1 cells transiently transfected with WT, p.G179V, p.R494Q, p.T860M and p.E1205G *TRPM7*. ^*^—*P* < 0.05, ^*^^*^—*P* < 0.01, ^*^^*^^*^—*P* < 0.001.

We transfected both cell lines with the four variants of *TRPM7* and recorded the current–voltage relationships. Due to its likely endogenous expression in both cell lines, we found *TRPM7* I–V relationships in all cells (Fig. 2). However, when analyzing grouped current density data, we found a significant reduction in current density in CHO-K1 cells when transfected with p.G179V (*P* < 0.05, Fig. 2A). We also saw a significant increase in current density at both +80 and −80 mV when expressing p.R494Q compared to WT–transfected cells (*P* < 0.001, Fig. 2A). When these experiments were repeated in HEK293 cells, we found that both p.G179V and p.T860M current density was markedly reduced compared to WT *TRPM7* (*P* < 0.01) with a trend to reduction for E1205G (Fig. 2B). However, in comparison to CHO-K1 expression of p.R494Q, current density was equal between WT and p.R494Q expressing cells (*P* = 0.98, Fig. 2B).

### Reducing extracellular Mg^2+^ amplifies difference between WT and mutant *TRPM7* channels

**Figure 3:**
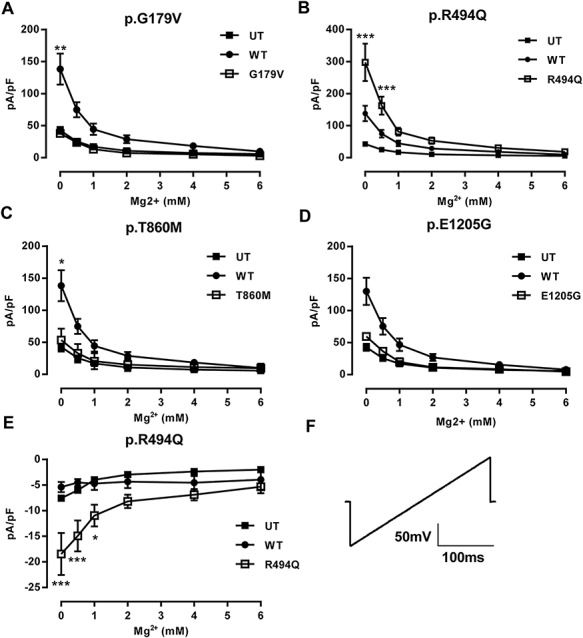
Stillbirth variants alter *TRPM7* current density at low concentrations of magnesium. (**A–D**) Average *TRPM7* current density recorded from transfected CHO-K1 cells at +80 mV in response to increasing concentrations of bath magnesium (*n* = 7–8). (**E**) Average inward current density (−80 mV) of cells transfected with p.R494Q *TRPM7*. (**F**) Voltage protocol used throughout these experiments. Holding potential was set at 0 mV and the protocol repeated every 5 s. ^*^ = *P* < 0.05, ^*^^*^ = *P* < 0.01, ^*^^*^^*^ = *P* < 0.001 vs WT.

We wanted to investigate whether the stillbirth mutations were affecting the *TRPM7* channel’s response to magnesium inhibition. We perfused transfected CHO-K1 cells with extracellular solution supplemented with increasing concentrations of magnesium from 0.5 to 6 mm after allowing current recordings to plateau. In these experiments, we found a significant decrease in current density at +80 mV in cells transfected with either p.G179V or p.T860M *TRPM7* compared to WT channels, but only at 0 mm Mg^2+^ (*P* < 0.01 and 0.05, respectively, Fig. 3A and C). However, when bath magnesium was increased, any difference in Mg^2+^-dependent inhibition between these variants and WT *TRPM7* was attenuated and eventually abolished. Cells expressing p.R494Q *TRPM7* showed a significant increase in current density at +80 mV compared to WT cells at both 0 and 0.5 mm Mg^2+^ (*P* < 0.001, Fig. 3B). We calculated the IC_50_ for WT-transfected cells at 0.62 mm Mg^2+^ with a Hill coefficient of −1.21. Further analysis of stillbirth variant-transfected cell IC_50_ and Hill coefficient values was not significantly different to WT *TRPM7* (Supplementary Material, Table S3). Interestingly, the inward current for this variant analyzed at −80 mV was increased compared to WT *TRPM7* at 0, 0.5 and 1 mm Mg^2+^ (*P* < 0.001, 0.001 and 0.05, respectively, Fig. 3E). Suggesting that despite physiological levels of magnesium, this variant increased both inward and outward ionic conductance compared to the WT *TRPM7* channel. These data also suggest that any difference in whole-cell *TRPM7* ion channel conductance are not the result of a variant influencing the channel’s response to magnesium inhibition but are more likely due to alterations in expression.

In summary, our electrophysiological data show that G179V and T860M result in a loss of channel function and R494Q a gain of function, which was particularly apparent in conditions of low extracellular Mg^2+^.

### Effect of variants on *TRPM7* protein and mRNA expression in heterologous cells

**Figure 4:**
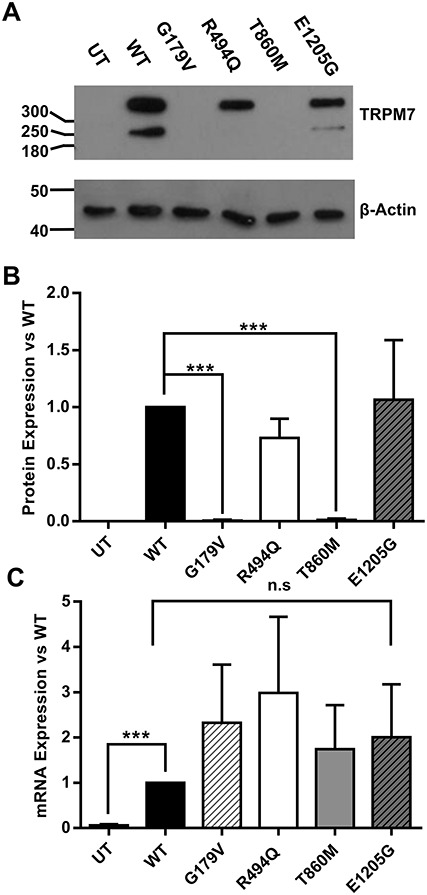
Stillbirth variants p.G179V and p.T860M reduce TRPM7 protein levels but leave transcription unchanged. (**A**) Whole cell lysates from HEK293 cells transiently transfected with wild-type TRPM7 and mutant vectors. PVDF membranes were initially blotted with anti-TRPM7 before stripping and reprobing with anti-β-actin. (**B**) Quantification of three western blots from whole-cell HEK293 lysates transfected with WT or stillbirth variant TRPM7 normalised to loading β expression before normalising to WT expression. C qPCR expression of TRPM7 from transfected cell lysates normalized to WT. Lysates were treated with DNase I for 15 minutes at room temperature before qPCR analysis. WT = wild-type, UT = untransfected. ***P < 0.001 vs WT.

To investigate whether this decrease in current density was due to altered protein levels, whole cell lysates were harvested from transfected cells and western blotting was performed. p.G179V and p.T860M *TRPM7* mutants did not express well in transiently transfected cells (Fig. 4A). Densitometry measurements showed a significant decrease in HEK293 cells transfected with either p.G179V or p.T860M *TRPM7* compared to WT *TRPM7*. However, when we investigated *TRPM7* and mutant mRNA levels in these cells using qPCR, the mRNA expression level was similar (Fig. 4C). To test whether this reduction in protein was due to rapid degradation, we harvested cell lysates after overnight treatment with the proteasomal inhibitor MG132. We observed an increase in p.R494Q-transfected cells compared to WT, and we may have able to detect low level expression of p.T860M *TRPM7* protein (Supplementary Material, Fig. S1).

### Confirming the presence of *TRPM7* current in hiPSC-derived cardiomyocytes

**Figure 5:**
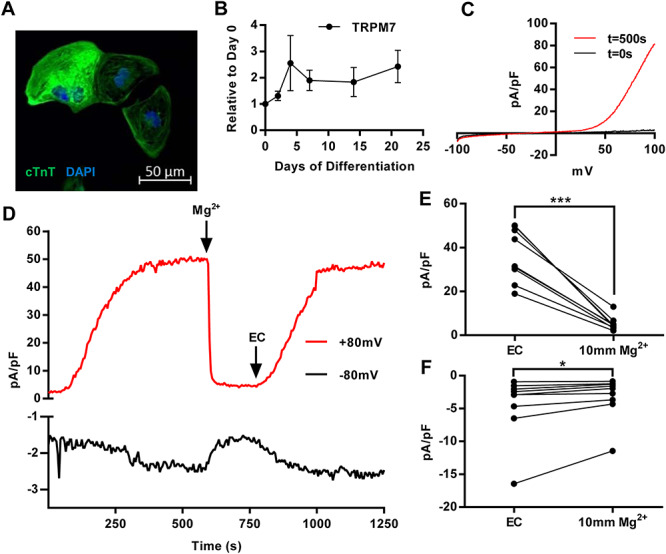
A *TRPM7*-like current can be found in 21-day-old hiPSC-derived cardiomyocytes. (**A**) Staining of hiPSC-CMs after 21 days of differentiation for cardiac troponin (cTnT) and DAPI. (**B**) qPCR analysis of *TRPM7* expression levels over the course of hiPSC-CM differentiation. *TRPM7* expression at each time point was normalized to GAPDH expression before being compared to *TRPM7* expression in undifferentiated hiPSC cells. (**C**) A representative *TRPM7* trace from whole-cell hiPSC-CMs. Initial current is shown (black) at initial current recordings before magnesium is removed from the bath, and a steady plateau is reached (red). (**D**) Time course recording of 22 day post-differentiation hiPSC-CM showing *TRPM7* whole-cell current run-up and transient inhibition of 10 mm Mg^2+^ at +80 mV (red) and −80 mV (black). (E) Paired individual cell current measurements at +80 mV peak during extracellular solution perfusion, cells were then treated with 10 mm Mg^2+^ over several minutes until current plateaued. (**F**) Inward current recorded at −80 mV in the same cells analyzed in (D), after current stabilization and after 10 mm Mg^2+^ treatment. ^*^ = *P* < 0.05, ^*^^*^^*^ = *P* < 0.001.

We used an *in vitro* model of cardiomyocyte generation using hiPSC cells to first ascertain *TRPM7* expression in human cardiac cells and to test the effect that reduced *TRPM7* expression may have on the electrophysiological properties of cardiomyocytes. We used an in-house hiPSC line (named HS1M) and differentiated the cells into cardiomyocytes using a previously published protocol (Supplementary Material, Fig. S2) (20). hiPSCs initially expressed high levels of TRA-1-60 and three key pluripotency markers, which rapidly decreased by 7 days of differentiation (20). Individual cells stained positively for cardiac troponin T (Fig. 5A). We observed variation in *TRPM7* expression during differentiation, with cardiomyocytes at day 21 expressing more than double that observed in hiPSC cells, but this was not significant (Fig. 5B). In contrast, pluripotency markers rapidly decreased as the cell differentiated (Supplementary Material, Fig. S2C).

Beating hiPSC-CMs were seeded onto gelatin-coated glass coverslips at low density at days 15–16 for single-cell patch-clamp analysis to detect *TRPM7* current at days 21–23. We found in all contractile cells subjected to whole-cell patch-clamp, typical *TRPM7* currents, which were detected after dialysis of cells with Mg^2+^ chelating intracellular solutions. Although initial break-in currents were minimal, outward currents at +80 mV were detected after 5–10 min (Fig. 5C, E and F). We perfused the bath solution with magnesium to confirm these currents were generated by *TRPM7* (Fig. 5D–F). We found that at both +80 and −80 mV, these currents were susceptible to magnesium inhibition (*P* < 0.001), which could be reversed following washout with standard extracellular solution.

### Mimicking *TRPM7* reduction using siRNA does not adversely affect action potential parameters in hiPSC-derived cardiomyocytes

**Figure 6:**
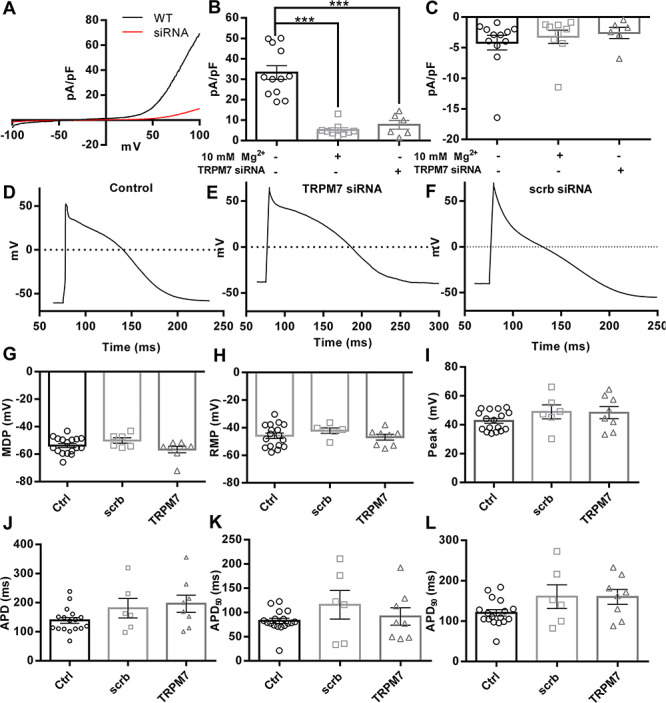
siRNA-mediated knockdown of *TRPM7* at day 16 of differentiation does not alter hiPSC-CM AP parameters. (**A**) Comparison of representative *TRPM7* currents after run-up in hiPSC-CMs with (black) or without *TRPM7* siRNA transfection (red). (**B**) *TRPM7* current measured at +80 mV following current run-up in non-targeting siRNA-treated hiPSC-CMs and the effect of magnesium addition or transfection with *TRPM7* siRNA. (**C**) Inward −80 mV current measured from the same cells as in (B). (**D**) Representative action potential trace of non-targeting siRNA-treated hiPSC-CMs. (**E**) Representative action potential trace of hiPSC-CM transfected with siRNA-*TRPM7*. (**F**) Representative action potential trace of hiPSC-CM transfected with siRNA-scrb. (**G**) Maximal diastolic potential. (**H**) Resting membrane potential. (**I**) Peak voltage following action potential triggering. (**J**) Time to return to RMP or full action potential duration in hiPSC CMs. (**K** and **L**), APD50 and APD90 measurements from the same cells in (J). All recordings were done in 20- to 23-day-old hiPSC-CMs at room temperature. Scrb = Scrambled. ^*^^*^^*^ = *P* < 0.001 vs control.

To mimic possible haploinsufficiency of *TRPM7* in developing cardiac cells due to p.G179V and p.T860M mutants, we used siRNA to reduce mRNA levels. To patch-clamp cells that had taken up *TRPM7* siRNA, we co-transfected siGLO. Whole-cell patch clamp analysis showed markedly reduced outward currents at +80 mV (*P* < 0.001, Fig. 6A and B) in cells transfected with *TRPM7* siRNA. On average, this led to a 25.5 pA/pF reduction in current density, a similar reduction to that seen in 10 mm Mg^2+^ treated cells (28.1 pA/pF). Perhaps due to their small initial size (<5 pA/pF), inward currents at −80 mV were not significantly different between control cells, those perfused with 10 mm Mg^2+^ or those transfected with *TRPM7* siRNA (Fig. 6C). RT-qPCR analysis of these cells found a significant decrease in *TRPM7* mRNA levels in siRNA-transfected populations (Supplementary Material, Fig. S3). After confirming *TRPM7* knockdown, we recorded triggered action potentials in a separate set of experiments (Fig. 6D and E). To establish whether there were any deleterious electrophysiological effects caused by reducing *TRPM7* ion channel expression, we analyzed action potential morphology (Fig. 6G–L). We found that there were no statistical differences in action potential duration, minimum diastolic potential, resting membrane potential and peak voltage amplitude in the action potentials of cells transfected with scrambled or *TRPM7*-targeted siRNA and untransfected cardiomyocytes.

## Discussion

Almost half of stillbirths go unexplained, despite extensive post-mortem analysis. In this study, we present data from previously sequenced and predicted damaging *TRPM7* genetic variants found in unexplained stillbirth cases (5). There is extensive research studying ion channelopathies that cause sudden cardiac death in young adults; however, there is little evidence of harmful ion channel variants in stillbirth (21). *TRPM7* is required for mammalian cardiac development, and genetic variation within the gene has been linked to a prolonged QT interval (7,15). To investigate the role these variants may play in perturbing the function of the *TRPM7* ion channel, we used heterologous expression and hiPSC-CMs to study channel function, magnesium response and expression.

Here, we show that three *TRPM7* variants modify ion channel function in comparison to the WT channel and identify *TRPM7* as an ion channel present in hiPSC-derived cardiomyocytes. First, we expressed and characterized the WT channel in HEK293 and CHO-K1 cells before analyzing the effects of the four *TRPM7* mutants. We report that p.G179V and p.T860M variants reduced outward currents significantly compared to the WT channel. Conversely, in a CHO-K1 specific manner, p.R494Q increased observed current density at both +80 and −80 mV. This increase remained significantly different despite inhibition with 1 mm Mg^2+^.

Protein expression data confirmed that decreased current density caused by the p.G179V and p.T860M variants was due to reduction in protein expression. In the case of p.T860M, this was at least in part due to rapid proteasomal degradation. It has been shown that *TRPM7* is required for proper embryonic development and cardiogenesis in mice (15,16). Targeted cre-mediated knockdown of *TRPM7* before E10 leads to congestive heart failure due to lack of myocardial compaction. Later deletion at E12.5 of gestation can lead to cardiomyopathy, heart block and impaired repolarization. Given the experimental data, it is feasible to postulate that a reduction in *TRPM7* function/expression during gestation could have an adverse effect on human cardiac development. Mechanistically, it is hard to determine the mechanisms that underlie this effect as *TRPM7*’s role in magnesium and zinc homeostasis, alongside control of apoptosis and gene expression is still under investigation (10,12,13,22). Reduced *TRPM7* expression may limit the cell’s ability to take-up divalent cations or hinder their expulsion—alongside adverse changes to a cell’s gene expression profile.

Recent work with cryo-EM microscopy has revealed the closed state structure of mouse *TRPM7* at the Ångstrom scale (23). These data show several interactions between the N-terminal domain and the inner plasma membrane, TRP domain or C-terminal domain. Non-synonymous variants found within these structural motifs may destabilize the protein, resulting in misfolding and degradation.

Using a hiPSC-derived model, we demonstrate that *TRPM7* can be found in immature human cardiomyocytes 21 days post differentiation. Analysis of temporal *TRPM7* expression during cardiomyocyte differentiation showed a gradual increase in mRNA, alongside the reduction in pluripotency factors. Single-cell patch-clamp revealed that these cells possess relatively large quantities of *TRPM7* present at the plasma membrane, with mean outward currents of (~33.3 pA/pF). Both treatments with Mg^2+^ and siRNA transfection significantly reduced these currents, strongly suggesting their identity as *TRPM7*. There is evidence to suggest that *TRPM7* can regulate cardiomyocyte ion channel gene expression, alongside acting as a cation channel in its own right (12,15,16). However, when we mimicked possible *TRPM7* haploinsufficiency, we could not detect an effect on action potential morphology. This may be due to the temporal aspect indicative of the *TRPM7* knockout phenotype shown in mice to vary from lethal to mildly perturbed repolarization depending upon the gestational stage at which *TRPM7* is removed. A more severe effect may occur if siRNA to *TRPM7* was transfected during differentiation, and this is an area that warrants further *in vitro* experimentation to identify critical windows of *TRPM7* expression during hiPSC-derived cardiomyocyte maturation. It is notable that increases in *TRPM7* expression occur relatively early in hiPSC cardiac differentiation. It is an open issue whether *TRPM7* currents contribute significantly to the fetal cardiac action potential. Our experiments with siRNA suggest that this isn’t the case under physiological ionic conditions, particularly due to the inhibitory effect of intracellular Mg^2+^ present in the tissue. Alternatively, haploinsufficiency may have deleterious effects that are not solely related to effects on cardiac excitability but to effects on cell survival mediated by the kinase domain.

In summary, we found two variants in *TRPM7*, p.G179V and p.T860M, reduce ion channel current expression, which in the case of p.T860M is likely due to rapid degradation mediated by the proteasome. In addition, the p.R494Q *TRPM7* variant significantly increases *TRPM7* ion channel current, in a cell-type specific manner. For the first time, we report that a *TRPM7* current is present in hiPSC-CMs. However, siRNA inhibition at day 15–16, despite reducing *TRPM7* current, did not alter AP duration. We believe that *TRPM7* may play a key role in ensuring correct cardiac development of the fetus. *TRPM7* is the major Ca^2+^ channel in atrial fibroblasts, and up-regulation of these transients is reported in patients with atrial fibrillation (24). Significant increases in these Ca^2+^ influxes from p.R494Q *TRPM7* in the developing atrial myocardium could predispose developing fetal hearts to atrial fibrillation. In a similar fashion, a reduction in *TRPM7* current may also predispose to cardiac arrhythmia. For example, it is known that both long and short QT syndromes arising from loss and gain of function mutations, respectively, in KCNQ1 can result in sudden cardiac death in afflicted families (25). Therefore, any changes to *TRPM7* abundance during early cardiogenesis may have severe downstream effects later in gestation, potentially creating a pro-arrhythmic cardiac environment. Linking causation with genetic variation is inherently difficult, but functional assays of potential impairment of protein function can contribute significantly to assessing potential pathogenicity (26). Our data indicate that inherited or *de novo TRPM7* mutations may increase the risk of unexplained stillbirth and this warrants further attention.

## Materials and Methods

### Cell culture, plasmids and transfection

Human Embryonic Kidney-293 cells (HEK293) were a gift from Professor Lily Jan (Howard Hughes Medical Institute, San Francisco, USA). Chinese Hamster Ovary-K1 (CHO-K1) cells were obtained from the European Collection of Authenticated Cell Cultures (supplied by Sigma—85 051 005). HEK293 and CHO-K1 cells were cultured in minimum essential medium (31 095 029, Gibco, ThermoFisher) and Ham’s F12 medium (N6658, Sigma-Aldrich), respectively. Both media were supplemented with 10% FBS (10 500 056, Gibco, ThermoFisher) and 100 units/ml of penicillin and 100 μg/ml of streptomycin (15 140 122, Gibco, ThermoFisher).

A pcDNA4/TO plasmid containing the *TRPM7* gene (RefSeq NG_021363.2, NM_017672.6), and a tetracycline repressor plasmid, pcDNA6/Tet Repressor were both kind gifts from Professor Schmitz (11). An eGFP plasmid (Clontech) was used to identify successfully transfected cells. Transfection was carried out using FuGENE^®^ HD (E2311, Promega) or NovaCHOice^®^ (72622-3, Merck) as per manufacturer’s instructions. Briefly, reactions of *TRPM7* vector and Tet Repressor plasmid were mixed with the transfection reagent in 37°C Opti-MEM^®^ reduced serum media. After 5–6 h, transfection complexes were removed and fresh media added. Precisely 24 h later, growth media was changed, this time supplemented with 1 μg/ml of tetracycline (87 128, Sigma-Aldrich). Cells were harvested/analyzed 24–48 h after tetracycline induction.

### Patch-clamp electrophysiology

Whole-cell patch-clamp recordings were made at room temperature (21–25°C), using a multiclamp 700B and digitized using a Digidata 1550B (Molecular Devices). The internal pipette contained (in mm) 145-Cs-methanesulfonate (CsSO_3_CH_3_), 8 NaCl, 10 HEPES and 10 EGTA; pH 7.2 with CsOH. The extracellular solution contained (in mm) 145 NaCl, 5 KCl, 2 CaCl_2_, 10 glucose and 10 HEPES (pH 7.4 with NaOH). For measuring *TRPM7* current in hiPSC-CMs, the internal pipette solution contained (in mm) 120 L-aspartic acid, 20 CsCl, 2.5 EGTA, 2.5 EDTA, 10 HEPES, 120 CsOH and 5 Na_2_GTP (pH 7.2 with CsOH). Extracellular solution contained (in mm) 135 NaCl, 5.4 CsCl, 10 HEPES, 10 glucose, 0.1 CdCl_2_ and 1 CaCl_2_ (pH 7.4 with NaOH). To record action potentials, internal pipette solution contained (in mm) 110 K-gluconate, 20 KCl, 10 HEPES, 0.05 EGTA, 0.5 MgCl_2_, 5 Mg ATP, 0.3 Na_2_-GTP and 5 Na_2_ phosphocreatine (pH 7.4 with KOH). Extracellular solution for action potential recordings (mm): 140 NaCL, 2.7 KCl, 1 MgCl_2_, 2 CaCl_2_, 0.5 Na_2_HPO_4_ and 5 glucose (pH 7.4 with NaOH). Only cells with membrane resistance >800 MΩ and access resistance <10 MΩ were used in analysis.

Pipettes were backfilled with intracellular solution to resistance 2–3 MΩ. Pipette tips were coated with SigmaCote to reduce pipette capacitance (SL2, Sigma-Aldrich). Series resistance was set to 70% compensation using the in-built amplifier circuitry. After whole-cell configuration was established, recording began immediately to observe current run-up. We used a ramp voltage protocol to measure *TRPM7* current: the holding potential was set at 0 mV before lowering to −100 mV and then raised to +100 mV over 250 ms before returning to 0 mV. For time-course analysis experiments, this was measured every 5 s from electrical access being established through the cell. Data were analyzed using Clampfit 10.5 (Molecular Devices).

In hiPSC-derived cardiomyocytes (hiPSC-CMs), we only analyzed triggered action potentials (APs) from cells with spontaneous action potentials. APs were triggered using a current pulse (between 400 and 800 pA) at a frequency of 1 Hz. To calculate action potential duration, the time (in ms) was calculated as the time it took for membrane potential to return to 50 and 90% (APD_50_ and APD_90_) of the resting membrane potential after current trigger. Cells were paced for 50 s before recording the final 10 action potentials. These results of the final 10 traces were combined to give an ‘average’ action potential for every cell. We did not analyze cells according to ventricular-like or atrial-like categorizations due to a limited number of cells.

### Protein harvesting, SDS-PAGE and western blotting

HEK293 cells were transfected at ~ 70% confluence as previously mentioned and harvested 24 h after tetracycline induction. Cells were washed with PBS^+^ (0.1 mm CaCl_2_ and 1 mm MgCl_2_) before a 30-min incubation on ice with NP-40 lysis buffer containing protease inhibitors (11 816 170 001, Sigma-Aldrich). Lysates were scraped into pre-chilled tubes and cleared by centrifugation at 10 000*g* for 10 min at 4°C. Amount of 20 μg of protein was loaded for each well into 6% polyacrylamide gels. Resolved proteins were transferred to PVDF membranes overnight and then blocked for 1 h in PBS-T with 5% milk powder or 1% BSA (A2153, Sigma-Aldrich). Membranes were then incubated with either anti-*TRPM7* (sc-271 099, Santa Cruz) or β-actin (ab8226, Abcam) antibodies for 1 h at room temperature. Secondary antibodies were incubated for 1 h at room temperature before imaging with Amersham ECL western blotting detection reagent (GE Healthcare). Primary and secondary antibody details are listed in Supplementary Material, Table S2. ImageJ software was used to quantify *TRPM7* expression, which was normalized to loading control β-actin levels, and then all stillbirth variant expression was normalized to the WT value in the same gel. For proteasomal inhibition experiments, cells were treated with 5 μM MG132 (Sigma-Aldrich) for 16 h prior to harvesting.

### RNA harvesting and quantitative PCR

Total cell RNA was harvested using a RNeasy mini kit (Qiagen) as per manufacturer’s instructions either 24 h after tetracycline induction or at specific time points of stem cell differentiation. During harvesting of RNA, the column was treated with DNase I for 15 min at room temperature. RNA was diluted in nuclease-free water, and 50 ng used per qPCR reaction. All probes used are listed in Supplementary Material, Table S3. Data were analyzed using 2^−∆∆Ct^ method normalized to loading control and then normalized to WT. To calculate gene expression in hiPSC-derived cardiomyocytes, RNA levels were first normalized to the housekeeping gene GAPDH. All expression values were then normalized to the expression of the gene of interest in naïve iPSC cells prior to differentiation.

### Confocal microscopy

Cells were first washed with PBS^+^ to remove media and then fixed with 3.7% formaldehyde (Sigma-Aldrich) for 10 min at room temperature. Cells were permeabilized with PBS Triton X-100 0.1% for 10 min. We blocked cells in PBS-T 5% milk for 1 h and carried out primary anti cTnT incubation for overnight at 4°C. Fluorescent secondary antibody incubation took 1 h at room temperature. All antibody details are listed in Supplementary Material, Table S1. Nuclei were counter stained with DAPI and all slides stored at 4°C until imaging with an LSM710 confocal microscope (Zeiss).

### Human stem cell-derived cardiomyocytes

Healthy human-induced pluripotent stem cell line (named—HS1M) was generated and maintained as previously described (27). Cardiomyocytes were differentiated using a protocol based upon previous work published by Burridge *et al.* (20). In brief, hiPSCs were seeded at 150 000 cells/cm^2^ in a 24-well plate and allowed to reach ~ 80% confluence over 48 h. Media was changed to ‘CDM3’ (RPMI1640 (Thermofisher) supplemented with 500 μg/ml *O. sativa*-derived recombinant human albumin (Sigma-Aldrich), 213 μg/ml L-ascorbic acid 2-phosphate (Sigma-Aldrich)) with 6 μM CHIR99021 (Sigma-Aldrich). After 48 h, media was changed to CDM3 supplemented with 2 μM C59 for a further 2 days before regular CDM3 media changes every 2–3 days. For patch-clamp analysis, at 15 days post-differentiation, cells were treated with TrypLE Express (Thermofisher) for 10 min at 37°C and seeded onto glass coverslips at a density of 10 000 cells/cm^2^.

For *TRPM7* siRNA knockdown experiments, 48 h before patch-clamp analysis, adhered cells were transfected with either SMARTpool: ON-TARGETplus *TRPM7* targeting siRNA or a scrambled control (L-005393-00-0005 or L-006233-00-0005, respectively, Dharmacon). DharmaFECT transfection reagent 1, 2.5 μl/well (T-2001-02, Dharmacon) was used to transfect 2.5 μl/well of 2 μM siRNA.

### Statistics

All data were analyzed with GraphPad Prism 6. Grouped data were tested with one-way ANOVA, using Dunnett’s multiple comparison to test variants vs WT unless otherwise stated. For individual analysis of two groups, unpaired *t*-tests were used to calculate statistical differences. When comparing two independent variables, two-way ANOVA with Sidak’s multiple comparison test was used. All group data are presented as mean ± S.E.M. Significant *P* values are denoted as ^*^ = *P* < 0.05, ^*^^*^ = *P* < 0.01 and ^*^^*^^*^ = *P* < 0.001.
